# Implementation and Validation of a Novel and Inexpensive Training Model for Laparoscopic Inguinal Hernia Repair

**DOI:** 10.3389/jaws.2022.10305

**Published:** 2022-03-23

**Authors:** Andres Hanssen, Diego A. Hanssen, Rafael A. Hanssen, Sergio Plotnikov, Jose Haddad, Jorge E. Daes

**Affiliations:** ^1^ Surgery Department Clínica Iberoamérica, Universidad Metropolitana de Barranquilla, Barranquilla, Colombia; ^2^ Department of Surgery, Bronx Care Health System, Albert Einstein ICAHAN School of Medicine, New York, NY, United States; ^3^ Wilhelmsburg Groß-Sand Hospital, Chirurgie, Germany; ^4^ Instituto Medico La Floresta, Caracas, Venezuela; ^5^ Minimally Invasive Surgery Department, Clínica Portoazul, Barranquilla, Colombia

**Keywords:** education, laparoscopic, inguinal hernia, training model, anatomy

## Abstract

**Purpose:** The aim of this study was to develop and validate a reproducible low-cost model useful for the development and acquisition of skills and competencies required for endoscopic hernia repairs.

**Methods:** Ten general surgery residents (PGY3) were instructed to construct the model and perform the maneuvers necessary for the simulation of laparoscopic inguinal hernioplasty by the *trans*-abdominal pre-peritoneal (TAPP) technique. They practiced for 4 weeks in the model, and the time required to perform simulated hernioplasty by the laparoscopic TAPP technique in the initial session was compared to the time required after 4 weeks of training.

**Results:** The time required to perform the exercise was significantly lower than in the initial session (*p* < 0.01). The time required by residents to complete the exercise in the initial session was significantly longer than that used by expert surgeons in the same task (*p* < 0.01), and although a significant difference persisted, this difference was substantially reduced to 3.60 min after the residents completed 4-week training in the model (*p* < 0.01). An independent expert, blinded to the level of training of the person who performed the exercise, could recognize all residents as novices and all experienced surgeons as experts in the initial session of the exercise with the model, but after 4 weeks of training, they did not recognize 4 of the 10 residents as novices (*p* < 0.05).

**Conclusion:** The routine implementation of training in this model could be very useful in the laparoscopic inguinal hernioplasty teaching-learning process.

## Introduction

Since the last decades of the 20th century, surgery has undergone progressive changes, moving toward the increasingly frequent use of minimally invasive surgical methods, which have in some cases displaced the conventional or “open” surgery techniques, and have now become the standard to treat many pathologies in the general surgery field. These methods increasingly demand mastery of new technologies. Thus, minimally invasive surgery has become an effective therapeutic option to treat various surgical conditions, including inguinal hernias (1), which makes training and acquisition of skills and competencies for this type of procedure vital (2,3). The speed of adoption of laparoscopic techniques for inguinal hernia repairs has been very slow and the adoption rate is still quite low (between 15% and 20% in the United States) (4), being lower in Latin American countries. In general, the prolonged and difficult learning curve is one of the factors that has conditioned these low adoption rates (5), which makes the development and implementation of courses, resources, models, and programs for training in these techniques imperative. Simulation is a valuable resource, which could play an important role in this process without implying potential risks for the patient, and without prolonging the operating times (1). For such reasons, the availability of a low-cost training model, which faithfully reproduces the endoscopic anatomy of the inguinal region and its possible variations and allows training and acquisition of the skills and abilities required in laparoscopic inguinal hernioplasty would be very useful (4). The advent of minimally invasive surgery techniques has created the need to acquire unique and specific motor skills for these types of procedures. That is why a whole new pattern of surgical education has emerged, in which many skills and abilities are developed in models and simulators in order to shorten the learning curves in a safe, controlled, and affordable environment, where the trainees’ progress can be monitored.

Hernia is a common pathology, with an estimated incidence of 5–15% (6). Of all hernias of the abdomen, about 75% are inguinal hernias. Inguinal hernia repairs are among the most frequently performed surgeries worldwide (7). Despite obvious cosmetic advantages, and a decrease in the time needed for the resumption of the patient’s daily activities, the adoption of laparoscopic inguinal hernioplasty by general surgeons has been much slower than for other procedures such as laparoscopic cholecystectomy (4). The reasons for this are varied, but include the need to be familiar with a perspective of the pelvic and inguinal anatomy different from the perspective most surgeons feel comfortable with during hernia repairs by conventional open techniques. The use of simulated procedures in inanimate models represents a very important tool in the training of minimally invasive procedures, in such a way that simulation has become an important element of training in today’s surgical world (4). Many publications have shown the relevance of training based on simulation models and the possible transfer of the skills and abilities learned under this modality in the actual surgical performance (8–11). In the aviation field, there are numerous publications, and vast experience, in the use of simulation to train the various actors of aeronautical activities, especially pilots, and the usefulness of subjecting personnel in training to the simulated environment, as well as to various circumstances which could arise during the performance of the actual activity (12, 13). In the last decade, some authors have proposed the implementation of simulation models in laparoscopic inguinal hernioplasty training, emphasizing specific technique maneuvers such as mesh placement and fixation (14, 15). Kurashima et al. developed a low-cost model for inguinal laparoscopic hernioplasty training at McGill University in Montreal, Canada that proved to be useful in the development of skills to perform all the necessary steps in laparoscopic inguinal hernia repairs (McGill Laparoscopic Inguinal Hernia Simulator MLIHS) (3). This model allows trainees to modify the simulated anatomy, emulating the different possible types of hernia. This publication establishes that simulation in this type of model can play an important role in shortening the learning curve of these procedures and increase the number of surgical residents with sufficient preparation to perform these interventions. Nishihara and collaborators have also published the validation of a similar model based on 3D printing, very similar to the real human anatomy, which uses mainly textile materials (14).

The creation of a training model which reproduced the endoscopic view of the inguinal region in the most precise way possible was proposed, using tissues of animal origin that resembled the consistency to the indirect touch obtained with laparoscopic instruments of the inguinal region wall and of the retro-pubic area. We propose that the resulting model allows the reproduction of the maneuvers or steps established by Daes and Felix in their article “Critical View of the Myo-pectineal Orifice” (15), and the 10 golden rules for safe inguinal hernia repair emphasized by Claus et al. (16) can be respected.

One of the most important motivations for developing this simulation tool was to get a low cost resource to introduce endoscopic hernia repair training with adequate mentoring for junior residents starting early in their educational process as recommended by current guidelines (17).

## Materials and Methods

**FIGURE 1:**
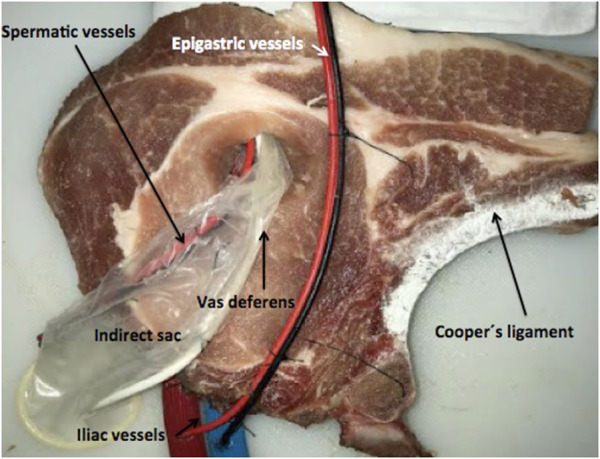
Indirect (Lateral) hernia model (Left side).

**FIGURE 2:**
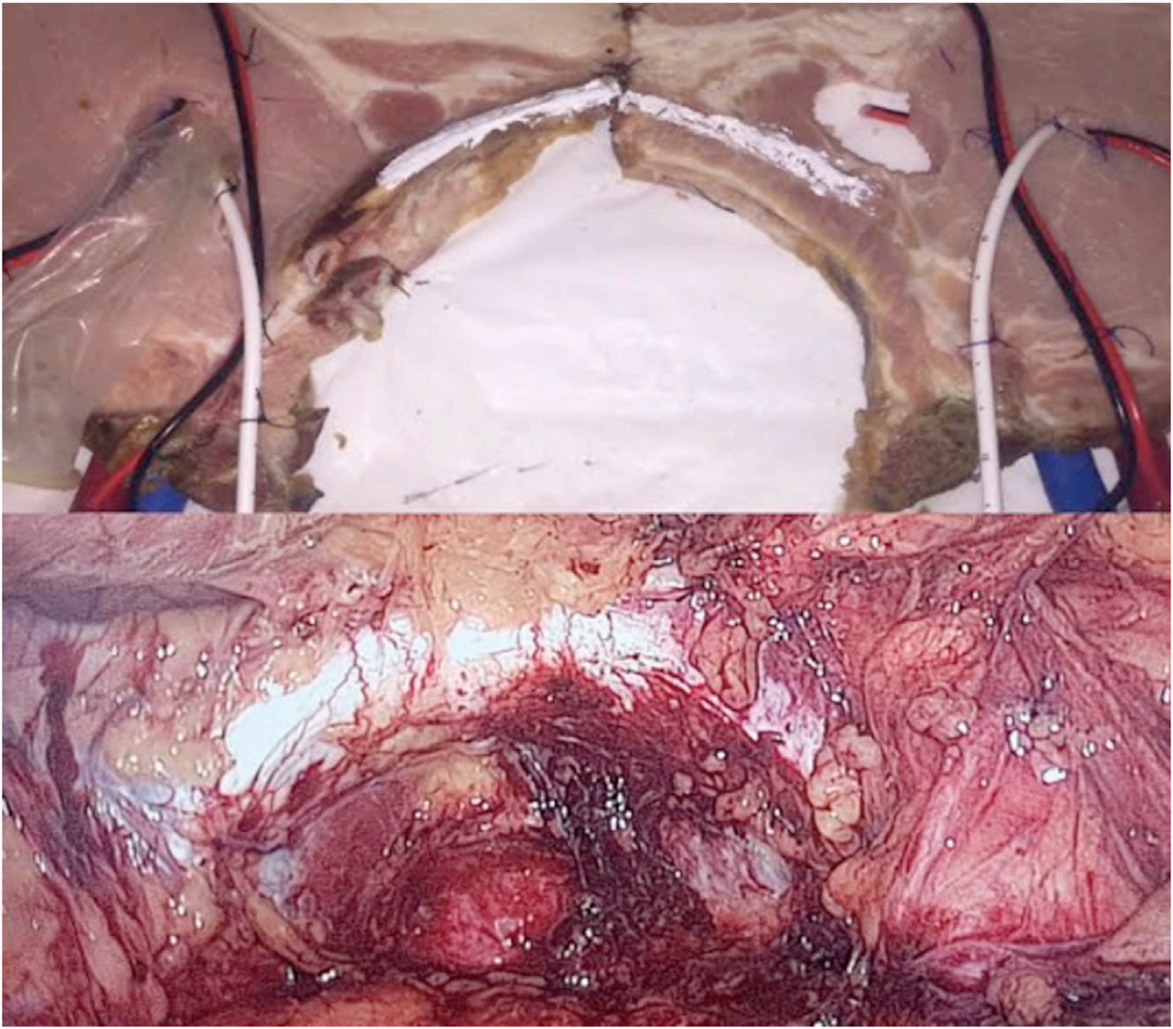
Hernia Model (both sides) and comparison with real anatomy.

**FIGURE 3:**
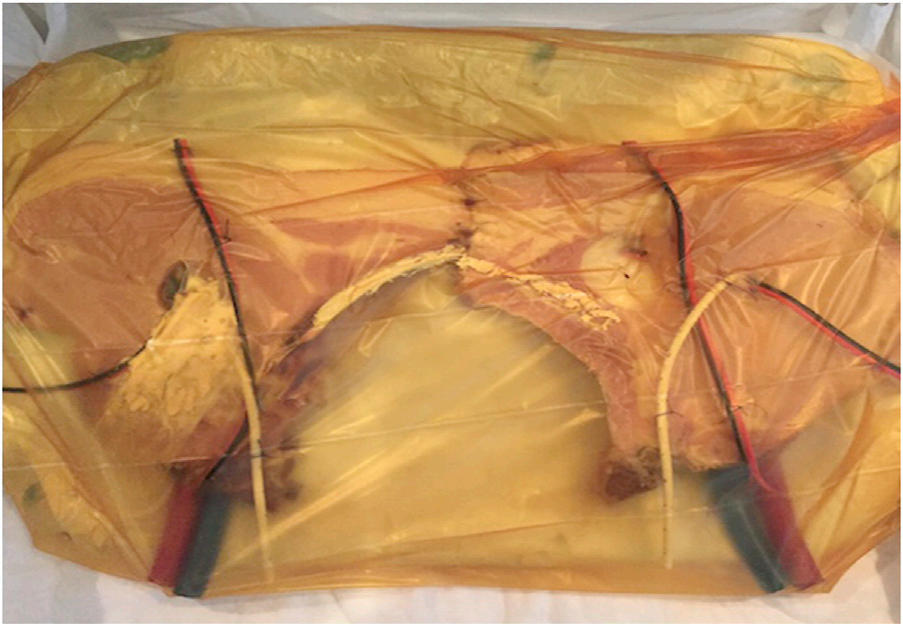
Hernia model (both sides) covered with simulated “peritoneum”.

We used pork chops that preserved the hemi-vertebra with its corresponding transverse process, which, when painted white, represented Cooper’s ligaments. Dual blue and red electric wires were used to represent the epigastric and the spermatic vessels, respectively. Two 30Fr polyethylene hoses were painted blue and red, respectively, to represent the iliac vessels, and the ends of the wires used to represent the epigastric vein and artery were inserted into them. A segment of a 9.5Fr caliber central venous catheter was used to simulate the vas deferens, a condom was used to represent the indirect hernia sac, and it was attached with silicone glue to the inguinal wall (the chop), as well as the cables and catheter used to simulate the elements of the spermatic cord. The distal portion of the condom, the cables used to represent the spermatic vessels, and the catheter used as a vas deferens were introduced into a hole made in the cutlet with a scalpel blade number 11 (removing a portion in a punch of 2 cm in diameter) to represent the enlarged deep orifice of the inguinal canal, assuming the anatomical configuration of an external or indirect oblique hernia (lateral), and preserving the relationship of this type of hernias with the epigastric vessels (Figure 1). Two models constructed in the same way were placed side by side, in order to represent both inguinal regions (Figure 2), and the complete preparation was coated with a self-adhesive plastic field ioban ™ 6648 (3MA^®^ St. Paul, MN, United States) to mimic the peritoneum (Figure 3). The cost of materials was about 60,000 Colombian pesos or 20 US dollars. This preparation was properly placed and fixed inside an endo-trainer, equipped with interior lighting, and a video camera connected to a television monitor (Endo-trainer Dr. ET, Servitroner Bogotá Colombia).

Ten general surgery residents in their third year (PGY·3) were instructed to prepare the model and to perform the necessary maneuvers for the simulation of laparoscopic inguinal hernioplasty by the pre-peritoneal transabdominal technique (TAPP) after watching a didactic video. The 10 residents practiced five maneuvers in the model for 4 weeks (with an average of 15 h in this period), namely (1) creation of the peritoneal flap (2); dissection of the pre-peritoneal space, detaching the simulated peritoneum (self-adhesive plastic drape) from the inguinal wall (represented by the pork chop) until obtaining the critical view of the myopectine orifice (3); treatment of the indirect hernia sac, by dissecting the condom that simulates the sac until it is completely separated from the elements of the spermatic cord, surrounding the sack and twisting it on its axis, ligating it using intra-corporeal suture and knotting (inside the endo-trainer), and sectioning it with endo-scissors (4); introduction, deployment, and fixation of a 10 × 12 cm Polypropylene mesh, using titanium helical mechanical fasteners (Protack ™ Medtronic ^®^), placing two of these fasteners in the soft tissue immediately above the transverse process of the cutlet (Cooper’s ligament), and three at the upper edge of the mesh, above the hypothetical line that connects both anterior and superior iliac spines; and (5) peritoneal flap closure, using a monofilament suture with intra-corporeal knotting. Residents performed an average of 25 simulated hernia repairs (20–27) during the training period of 4 weeks. Each model was used several times and re-arranged after completion of the maneuvers or tasks. The models were re-used after being preserved under refrigeration for periods up to 24 h.

The training sessions were videotaped using a Medicapture 200 ™ device (MediCapture Inc. Plymouth Meeting, PA, United States). The Kolmogorov–Smirnov test was used to assess the distribution of resident and expert time data to perform the maneuvers (initial and final). Student’s t-test was used to compare the times required by residents, before and after the training period, and those times to the time required by five “expert surgeons” (with over 100 laparoscopic hernia repairs performed) during the same tasks. Levene’s test was employed to compare the variances of both groups, and Fisher’s test was used to compare the standard deviation between the times before and after the training period. Analysis of variance (ANOVA) was also used for assessing differences between the times in the groups. A blinded independent observer reviewed the videos of the experts (10 videos) and videos of the recorded procedures performed by the residents at the initial session and after the completion of the training period. Kappa’s coefficient was calculated to establish the independent blinded expert viewer’s accuracy of judgement on the training level of the performer in each video.

A second blinded expert surgeon reviewed the video recorded sessions of experts and residents, without knowing to whom every specific video belonged, or if the video corresponded to the initial session, or after completing the training period. A score from 1 to 5 points was assigned to qualify the performance in each of the tasks or maneuvers in the model. The blinded expert was instructed to qualify each task as follows: 1—unacceptable, 2–poor, 3–acceptable, 4—good, and 5–excellent. The evaluation took into account (1) instrumental conflict (2), precision and economy of movements (3), tissue handling (4), proficiency for knot tying, and (5) adequate mesh and tacks position. The average (mean) scores of each resident in the complete procedure (including the five maneuvers) was registered at the initial session and after completing the training period; expert surgeons’ videos were also qualified using the same score and criteria. ANOVA was used in the comparison of the proficiency scores obtained by the residents at the initial session and after completing the training period using the model and with the expert surgeons’ scores.

## Results

**FIGURE 4:**
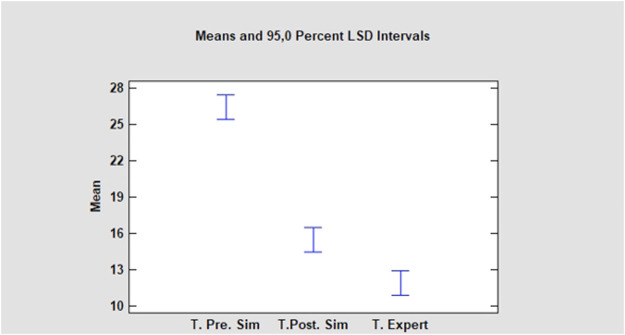
Least Significant Differences (LSD) Intervals. Resident Times (in minutes) at the initial session (T. Pre. Sim). After the training period (T. Post. Sim) and Expert surgeons time (T. Expert).

A significant decrease in the execution time of the maneuvers by the residents was observed after the training period, since the average time before the training was 26.40 min (22–30) with a standard deviation of ±2.37 min and the average time after training was 15.50 min (13–21) with a standard deviation of ±2.59 min. There was a substantial reduction in the average time needed by the residents to complete the procedure after the training period, with an average difference of 10.90 min (Figure 4).

The results of Student’s t-test were t = 15,099, with nine degrees of freedom and a bilateral significance = 0.000 (*p* < 0.01).

Confidence intervals of 95.0% were defined for the average time before training (T. Pre. Sim): 26.4 ± 1.69285 [24,7072; 28.0928] and the times after completing the 4 weeks of training (T. Post. Sim): 15.5 ± 1.85473 [13.6453; 17,3547]; obtaining for these confidence intervals a difference of means between the times (T. Pre.Sim and T. Post. Sim) assuming equal variances of 10.9 ± 2.33214 [8.56786; 13,2321].

Levene’s test showed a P value of 0.385225, confirming that there was no difference between the variances of the groups.

The average population difference between pre and post training times was statistically significant and was between 8.56 and 13.23 min with 95% confidence.

Fisher’s test showed an F value of 0.833058 and a P value of 0.790002 (>0.05). Showing no significant differences in the variability of the times before and after training, with 95% confidence.

Similarly, when comparing the experts’ times in performing the maneuvers to the time required by the residents to complete the tasks after the training period, a significant difference persisted, since the average time by the experts was 11.90 min (10–14) with a standard deviation of ±1.45 min and the average time by residents after training, as described above, was 15.50 min (13–21) with a standard deviation of ±2.59 min. But the average difference decreased to just 3.60 min (t = 3,933, *p* < 0.01). ANOVA showed an F-ratio of 118.57 and a P-value of 0.0000.

Regarding the assessment made by an independent observer on the exercises executed by the experts and by the residents after completing the training period using the model, the independent blinded observer recognized that a total of 14 exercises recorded on video were performed by experts, of which 10 really corresponded to experts, while the remaining 4 videos corresponded to residents with only 4 weeks of training using the model, but their exercises were not recognized as made by trainees.

The resulting value of the Kappa coefficient was +1 at the initial session before the training period and 0.6 after the 4 weeks using the model, with an approximate significance of 0.003, (*p* < 0.05).

Residents’ proficiency scores had an average of 1.9 points with a standard deviation of 0.42 at the initial session and 3.68 with a standard deviation of 0.27 after completing the training period.

**FIGURE 5:**
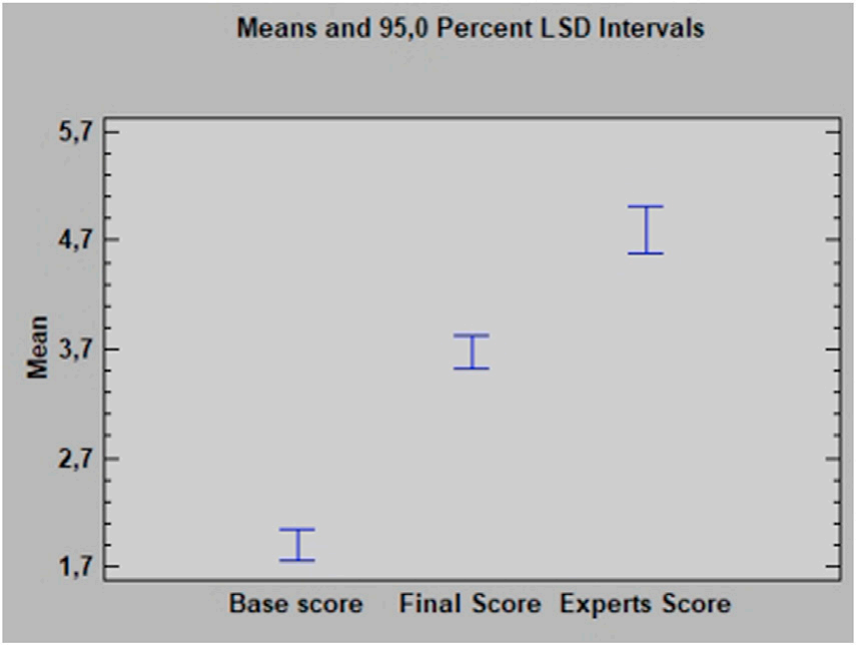
Least Significant Difference (LSD) Intervals. Residents’ Proficiency Scores (1–5 points) at the initial session (Base Score). After the training period (Final Score) and Expert surgeons’ Score.

Proficiency scores before and after the training period showed a normal distribution when the Kolmogorov-Smirnov test was applied. Expert surgeons’ scores also showed a normal distribution. ANOVA showed a P-value of 0.0000 and an F-ratio of 118.57 showing a significant increase of residents’ scores after the training period while still a significant difference between the novices’ scores after completing the training and the experts’ scores (Figure 5).

## Discussion

The increasing introduction of minimally invasive surgical techniques has created specific training needs for them. The observation of procedures performed by trained specialists or the completion of stages of interventions or complete procedures under the guidance of an expert teacher could require considerable time and a high number of procedures, without mentioning that this methodology could increase surgical times or represent risks for patients. Simulation has become an important tool for surgical training (4), and numerous publications have demonstrated the value of training in simulation models and the transfer of skills acquired in this way to surgical practice and performance in the operating room (5–8). Some publications have reported the implementation of training models for laparoscopic inguinal hernioplasty, but most of them have focused on the placement of the mesh and its fixation (11, 12). Kurashima et al. published the development of a low-cost inanimate model, built mainly with textile and synthetic elements, focused on training all the necessary steps to perform laparoscopic inguinal hernioplasty procedures (McGill Laparoscopic Inguinal Hernia Simulator MLIHS) (3). This model shares its versatility in terms of being able to modify the simulated anatomy, representing the various types of hernia, as well as representing the indirect hernia sac, and requiring the training and acquisition of the skills necessary to treat it with the one we present. Similarly, Nishihara and collaborators (14) reported the creation and validation of an ingenious training model for hernioplasty by the TAPP technique. This model was developed through the use of a 3D printer, it is quite similar to the real human anatomy, and uses various materials, mainly textiles (in particular, synthetic sponges) to simulate the laparoscopic aspect of the inguinal region. However, although the cost of the materials that represent the inguinal region structures is not high, the complete simulator is. In the model that we present, the placement of the trocars, and the simulation of the laparoscopic access are not performed, due to the use of the pre-existing ports in the endo-trainer. The use of biological materials, with tissue and bone, in combination with some synthetic elements allows a better correlation of the simulated element consistency, with those of the real anatomy, especially when the mesh fixation is simulated. Additionally, the MLIHS model requires several hours of preparation, while the time required by residents to prepare the one we report is on average 25 min, with the potential disadvantage, that by using biological tissues, its durability is considerably limited (they were only reconditioned and reused in a 24-h period, being preserved under refrigeration), but with the enormous advantage that the materials used in the model are very easily accessible in any supermarket or butcher shop, some hardware stores, and almost all hospitals. Likewise, any commercial or improvised endo-trainer can host the model without requiring additional costs.

The model we present can be arranged to simulate all types of hernias of the inguinal region. We decided to analyze the tasks or maneuvers needed to correct indirect hernias because of the relatively more complex nature of this kind of hernia and repair, but all types of hernias of the inguinal regions can be simulated in the model.

Regarding the fixation method used during the training period, we are aware that current guidelines only recommend using fixation in medial type 3 (large direct) hernias. However, there is no consensus about a “best” fixation method and surgeons’ preferences are recognized as the main selection criteria (17). Despite analyzing only indirect hernia repair tasks in our simulated inguinal hernia repair, we decided to include this fixation method (usually not necessary and recommended in this type of hernia) to familiarize trainees with the handling and use of the device and because of practical reasons (a single tacks applier allowed the participants to perform the complete procedure six times and the models were easy to re-arrange for re-use), but all invasive and non invasive fixation methods (glue, self-gripping meshes, tack sutures, etc.) can be taught and trained in our model.

Our results showed that despite having statistically significant differences between the times recorded by the experts compared to the residents in carrying out the exercise, the use of the training model in the period studied was effective for the development of useful skills in that context, and possibly in the learning of specific maneuvers necessary in laparoscopic inguinal hernioplasty, since with only 4 weeks of practical training, the difference between the average times to perform the exercise by novices differed from the times of expert surgeons to perform the same exercises in just 3.60 min. Two questions arise: What would be the result of this comparison if training time with the proposed model was significantly increased? At what point in the training would the times required by residents regularly match those of expert surgeons?

The evaluation of the possible effective transfer of skills and competencies, developed with training in our model, goes beyond the expectations of the present study, but represents an important motivation to study this impact in the future. The influence of training in models and simulation systems to improve performance in the operating room has been previously reported in some publications (7, 18, 19).

We used a scale score to assess competency, based on the judgement of an expert surgeon on the proficiency to perform the tasks in the model, showing a significant improvement over time using the model. Similarly, an expert surgeon, blinded to the training level of the performer of the tasks, reviewed the videos of the participants and made a judgment on the “quality” of the performance, trying to classify the video as belonging to a resident or to an expert surgeon, and this constitutes a “qualitative” assessment of the performance itself. Interestingly, the accuracy of the judgment was 100% at the beginning of the training period, but only 60% after completing the 4 weeks using the model. The performance in the model allowed the assessor to recognize and differentiate experts from beginners at the initial session, which is required to “validate” the instrument, but performance quality of the trainees changed during the period where they used the model, and this change probably can be attributed to the skills they acquired during the training sessions.

## Conclusion

We successfully built and implemented a low-cost training model for laparoscopic surgery of inguinal hernias with important anatomical fidelity in relation to the conformation of the human inguinal region from its intra-abdominal aspect. The proposed model allows the acquisition of useful skills and abilities in performing laparoscopic inguinal hernioplasties, and could represent an important tool in the learning of these interventions, shortening the learning curve without exposing patients to risks or prolonging surgical times. The preparation of the model before its use in the training sessions is useful to familiarize the resident or surgeon with the laparoscopic view of the inguinal region. Our model allows trainees to practice the most challenging maneuvers in laparoscopic inguinal hernioplasty, stimulating the development of necessary skills.

The routine implementation of training in this model could be very useful in the teaching-learning process of laparoscopic inguinal hernioplasty, especially in low-resource programs and developing countries with limited access to expensive sophisticated simulation systems.

## Data Availability

The raw data supporting the conclusions of this article will be made available by the authors, without undue reservation.
